# Comparison of skip-level ACDF vs. contiguous ACDF in the treatment of skip-level cervical degenerative disease: a systematic review and meta-analysis

**DOI:** 10.3389/fsurg.2026.1817062

**Published:** 2026-07-28

**Authors:** Juan Xie, Ling Zhu, Jianna Zhang

**Affiliations:** 1 Department of Emergency Medicine, West China Hospital, Sichuan University/West China School of Nursing, Sichuan University, Chengdu, China; 2 Disaster Medical Center, Sichuan University, Chengdu, China; 3 Nursing Key Laboratory of Sichuan Province, Chengdu, China

**Keywords:** adjacent segment degeneration, anterior cervical discectomy and fusion, cervical degenerative disease, noncontiguous, skip-level

## Abstract

**Background:**

The selection of surgical regimens for skip-level cervical degenerative disease (CDD) has long been controversial, with a core focus on whether intact intervertebral discs at intermediate levels should be preserved. This systematic review and meta-analysis compared the clinical and radiological outcomes as well as complication rates between skip-level anterior cervical discectomy and fusion (ACDF) and contiguous ACDF for skip-level CDD.

**Methods:**

This systematic review and meta-analysis was performed in accordance with the PRISMA 2020 Statement. The PubMed, Web of Science core collection, Embase (via Ovid), CNKI and WanFang databases were systematically searched from inception to November 30, 2025. This systematic review and meta-analysis was conducted via Review Manager 5.3.

**Results:**

Five studies with 116 patients in the skip-level ACDF group and 158 patients in the contiguous ACDF group were included. The surgical time [MD: −42.18, 95% CI (−50.91, −33.45), I^2^ = 72%] and intraoperative blood loss [MD: −28.89, 95% CI (−33.63, −24.14), I^2^ = 78%] were significantly lower in the skip-level ACDF group than in the contiguous ACDF group. No significant differences in the JOA score, C2-7 ROM, C2-7 SVA, T1 slope, complication rate or adjacent segment degeneration were detected between the two groups.

**Conclusion:**

Current evidence suggests that skip-level ACDF can reduce surgical time and intraoperative blood loss. Current evidence does not reveal a statistically significant increase in adjacent segment degeneration following skip-level ACDF. However, the available evidence remains limited.

**Systematic Review Registration:**

https://www.crd.york.ac.uk/PROSPERO/view/CRD420251243686, Identifier CRD420251243686.

## 1 Introduction

Smith GW and Robinson RA (1) initially introduced anterior cervical discectomy and fusion (ACDF) for the treatment of cervical degenerative disease (CDD) in 1958. With advancements in surgical techniques, cervical spine surgery has been characterized by increasingly diverse options, such as cervical disc replacement (2), anterior cervical corpectomy and fusion (3) and hybrid surgery (4), increasing the complexity of surgical decision-making. Despite the passage of more than six decades, ACDF has remained recognized as the gold standard for such conditions.

Skip-level CDD is a degenerative condition that primarily affects the intervertebral discs of two nonadjacent cervical spinal segments. It is characterized by the presence of one or more intervertebral discs with normal or near-normal structure and function between the pathological discs at the upper and lower ends of the affected region (5). Given that the incidence of contiguous multisegmental CDD is significantly greater than that of skip-level CDD, previous studies have focused primarily on the efficacy of anterior cervical surgery for contiguous multisegmental CDD (6). With the passage of time, however, skip-level CDD has attracted increasing attention in clinical practice.

While contemporary clinical practice for skip-level CDD strongly favors preservation of motion segments whenever feasible, there remains genuine clinical uncertainty regarding the optimal management when the intermediate segment demonstrates mild to moderate preexisting degeneration. The core clinical decision revolves around whether to preserve the intermediate intervertebral disc or extend the fusion to include this level (7).

Contiguous ACDF has historically been used for the treatment of multilevel CDD. However, extending the fusion construct to include a relatively intact intermediate segment is associated with well-documented increased risks of pseudarthrosis, bone graft nonunion, reoperation, and accelerated adjacent segment degeneration (4, 8, 9). For these reasons, contiguous ACDF is generally approached with caution in modern clinical practice.

To avoid these risks, the concept of skip-level ACDF, whereby only the pathological segments are fused while preserving the intermediate nondiseased disc, has gained widespread acceptance. Numerous clinical studies have demonstrated the safety and efficacy of this approach for skip-level CDD (10–12). Zhang et al. (13) compared zero-profile interbody fusion cages with traditional plate-cage constructs in 44 patients with skip-level CDD and reported that both techniques provided comparable satisfactory clinical outcomes and radiographic fusion rates. Baumann et al. (14) subsequently evaluated five different anterior surgical approaches for skip-level CDD and reported no significant differences in postoperative functional outcomes as assessed by the JOA score and NDI score. However, a major limitation of this study was that it did not include contiguous ACDF in the comparative analysis. Despite the growing acceptance of skip-level ACDF, concerns remain regarding the long-term fate of the preserved intermediate segment. Some studies have suggested that the altered biomechanical environment following skip-level ACDF may accelerate degeneration at the retained intermediate level (15–18), particularly when mild degenerative changes preexist. This potential risk remains the primary area of debate in the surgical management of skip-level CDD.

Given the aforementioned background, debates persist regarding the performance of skip-level ACDF and contiguous ACDF for skip-level CDD, and robust systematic review evidence is insufficient. This study hypothesizes that the two surgical approaches yield equivalent clinical and radiological outcomes as well as complication rates. Thus, we performed this systematic review and meta-analysis to compare the clinical and radiological outcomes as well as complication rates between skip-level ACDF and contiguous ACDF for skip-level CDD.

## 2 Materials and methods

### 2.1 Study registration and search strategy

The systematic review and meta-analysis were performed according to the PRISMA 2020 Statement (19). The registration number was CRD420251243686 at PROSPERO. The PubMed, Web of Science core collection, Embase (via Ovid), CNKI and WanFang databases were systematically searched from inception to November 30, 2025. The combinations of the following keywords were searched in the title or abstract: (anterior cervical discectomy and fusion OR ACDF) AND (noncontiguous OR non-contiguous OR skip-level OR skip level OR noncontinuous OR non-continuous). The complete search strategies are presented in Supplementary Table 1. No language restriction was applied. The references of relevant articles were also searched.

### 2.2 Inclusion and exclusion criteria

The screening was performed by two reviewers independently, and any disagreements were resolved by discussion with the third author. Studies that met the following criteria were included: (1) studies comparing the clinical and radiological outcomes as well as complication rates of skip-level ACDF vs. contiguous ACDF for skip-level CDD. (2) Randomized controlled trials (RCTs) and prospective and retrospective cohort studies were included. (3) The patients were clinically confirmed to have skip-level CDD. (4) The results of computed tomography or magnetic resonance imaging were consistent with the clinical symptoms. (5) No response to conservative treatment lasting for a minimum of 6 weeks. Studies that met the following criteria were excluded: 1) unrelated studies; 2) case reports; 3) biomechanical studies; 4) reviews; 5) noncomparative studies; 6) anterior cervical corpectomy; and 7) hybrid surgeries, such as ACDF + artificial cervical disc replacement (ACDR).

### 2.3 Data extraction

The following data were extracted by two authors separately: 1) first author, year of publication, institution and country; 2) age, sex and follow-up period of the patients; 3) methodological data; 4) operation time; 5) intraoperative blood loss; 6) JOA score; 7) C2-7 range of motion (ROM); 8) C2-7 sagittal vertical axis (SVA); 9) T1 slope; 10) adjacent segment degeneration; and 11) complication rate. Disagreements between the two authors were discussed and resolved with a third author.

### 2.4 Risk of bias assessment

Two independent authors assessed the risk of bias of the included RCTs using the Cochrane Risk of Bias 2.0 (RoB 2.0) (20) across five domains: randomization process, deviations from intended interventions, missing outcome data, outcome measurement, and selective reporting. Each domain was assigned a risk level of low, some concerns, or high risk on the basis of responses to the structured signaling questions. Two authors independently evaluated the risk of bias of all included nonrandomized cohort studies using the Cochrane ROBINS-I tool (21) across seven domains: confounding, participant selection, intervention classification, deviations from intended interventions, missing data, outcome measurement, and selective reporting. Each domain was graded as low, moderate, serious, or critical risk. Disagreements between the two reviewers were resolved via iterative discussion with a third senior author.

### 2.5 Statistical analysis

This systematic review and meta-analysis was conducted via Review Manager 5.3 (RevMan 5.3; Cochrane Collaboration). Adjacent segment degeneration and complication rates were assessed by relative risk (RR) and 95% confidence intervals (CIs). The operation time, intraoperative blood loss, JOA score, C2-7 ROM, C2-7 SVA and T1 slope were evaluated by the mean difference (MD) and 95% CI. Subgroup analysis was performed on the basis of the follow-up time. *P* < 0.05 was considered to indicate statistical significance. A random effects model was applied for all the parameters because clinical heterogeneity existed in almost all the clinical meta-analyses regardless of the I^2^ value (22). Subgroup analysis was conducted to explore the source of heterogeneity. Publication bias was assessed through a funnel plot when the number of included studies exceeded 10. The reliability of the results was evaluated via sensitivity analysis. When the number of studies we included exceeded 10, we evaluated the stability through meta-regression. The certainty of evidence was evaluated independently by two investigators in accordance with the Grading of Recommendations, Assessment, Development, and Evaluation (GRADE) guidelines (23).

## 3 Results

### 3.1 Search results

**Figure 1:**
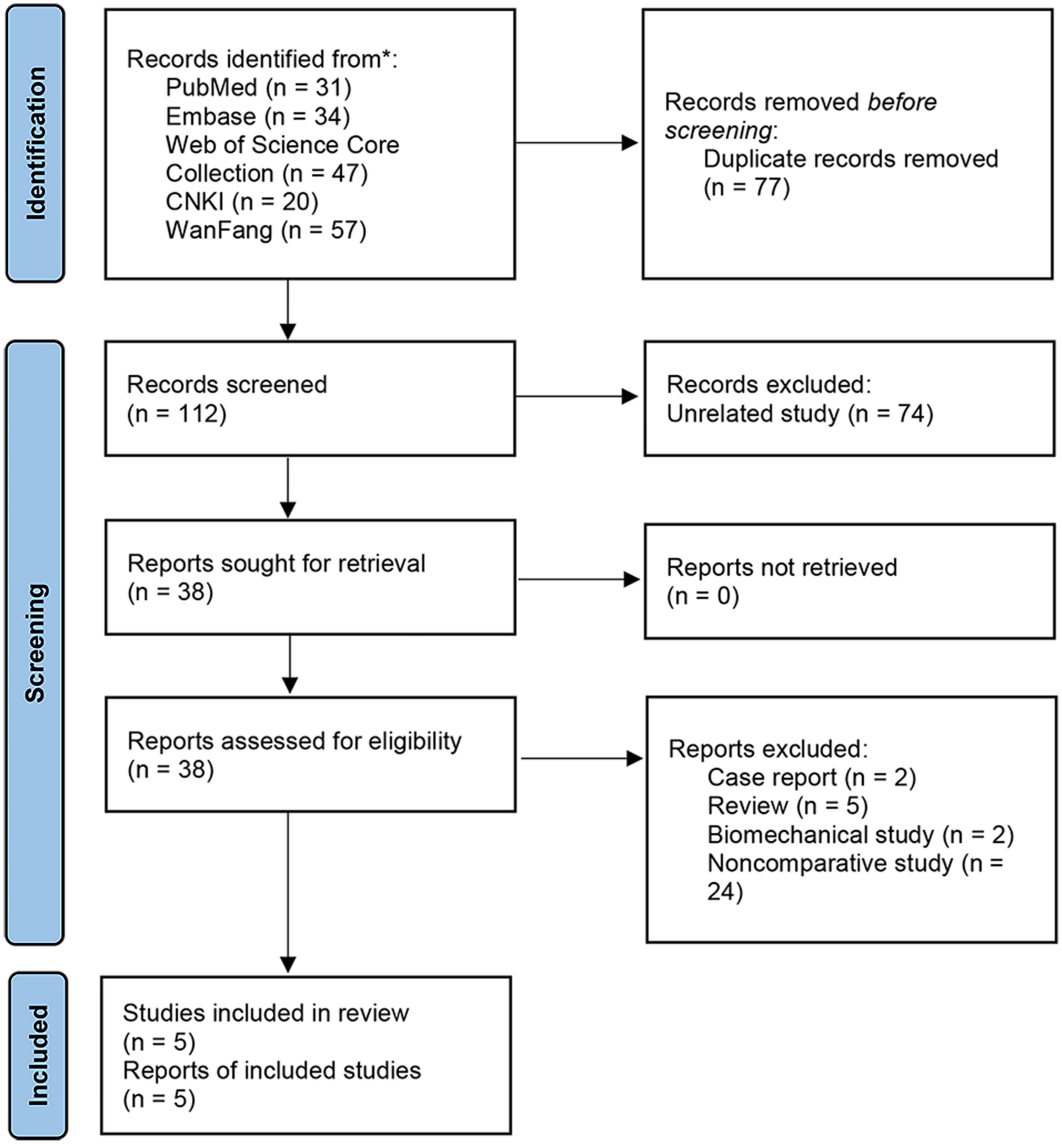
A flowchart of study selection.

A total of 189 studies were identified by searching the PubMed, Embase (via Ovid), Web of Science core collection, CNKI and WanFang databases. After the removal of duplicates, 112 studies were identified. Two case reports, 74 unrelated studies, 5 reviews, 2 biomechanical studies and 24 noncomparative studies were excluded. Five studies comparing skip-level ACDF vs. contiguous ACDF for skip-level CDD were included in this systematic review and meta-analysis (7, 24–27). The flowchart of study selection is shown in Figure 1.

### 3.2 Characteristics of the included studies and patients

Two randomized controlled trials and three retrospective cohort studies were identified. The basic information of the included studies is shown in Table 1. A total of 116 patients (81 males and 35 females) underwent skip-level ACDF, and 158 patients (118 males and 40 females) underwent contiguous ACDF. Among the included studies, the mean age of patients in the skip-level ACDF group ranged from 51.0 to 58.1 years, whereas that in the contiguous ACDF group ranged from 51.6 to 58.1 years. The mean follow-up duration of the included studies ranged from 12 to 24.67 months. Two studies reported the surgical level and skipped level. Among the 54 patients, C3–4/5–6, C4–5/6–7 and C3–4/6–7 skip-level ACDF was performed in 27 patients, 18 patients and 9 patients, respectively.

**Table 1:** Basic information of the included studies.

Study	Study design	Country	Sample		Mean age (y)		Gender (M/F)		Follow-up (Month)		Surgical level		Skipped level	Number of skipped level
			S	C	S	C	S	C	S	C	S	C		
Shah 2025 (10)	RCS	India	9	43	51.00 ± 3.43	51.6 ± 9.5	7/2	37/6	20.03 ± 10.53	24.67 ± 23.17	NR		NR	NR
Xie 2018 (24)	RCS	China	18	20	52.2 ± 10.1	54.7 ± 12.1	14/4	16/4	12 (6–36)		C3–4/5–6 (9) C4–5/6–7 (6) C3–4/6–7 (3)	C3–6 (11) C4–7 (9)	C4–5 (9) C5–6 (6) C4–6 (3)	One-level: 15 Two-level:3
Chen 2020 (25)	RCT	China	23	23	58.1 ± 3.2	58.1 ± 3.3	15/8	16/7	12		NR		NR	NR
Peng 2022 (26)	RCS	China	36	42	51.82 ± 4.72	52.3 ± 4.96	27/9	30/12	13 (8–19)		C3–4/5–6 (18) C4–5/6–7 (12) C3–4/6–7 (6)	C3–6 (24) C4–7 (18)	C4–5 (18) C5–6 (12) C4–6 (6)	One-level: 30 Two-level:6
Ding 2025 (27)	RCT	China	30	30	51.83 ± 1.21	51.76 ± 1.54	18/12	19/11	12		NR		NR	One-level: 21 Two-level:9

RCS, retrospective cohort study; RCT, randomized controlled trial; S, skip-level ACDF; C, contiguous ACDF; NR, not reported.

### 3.3 Quality assessment

**Figure 2:**
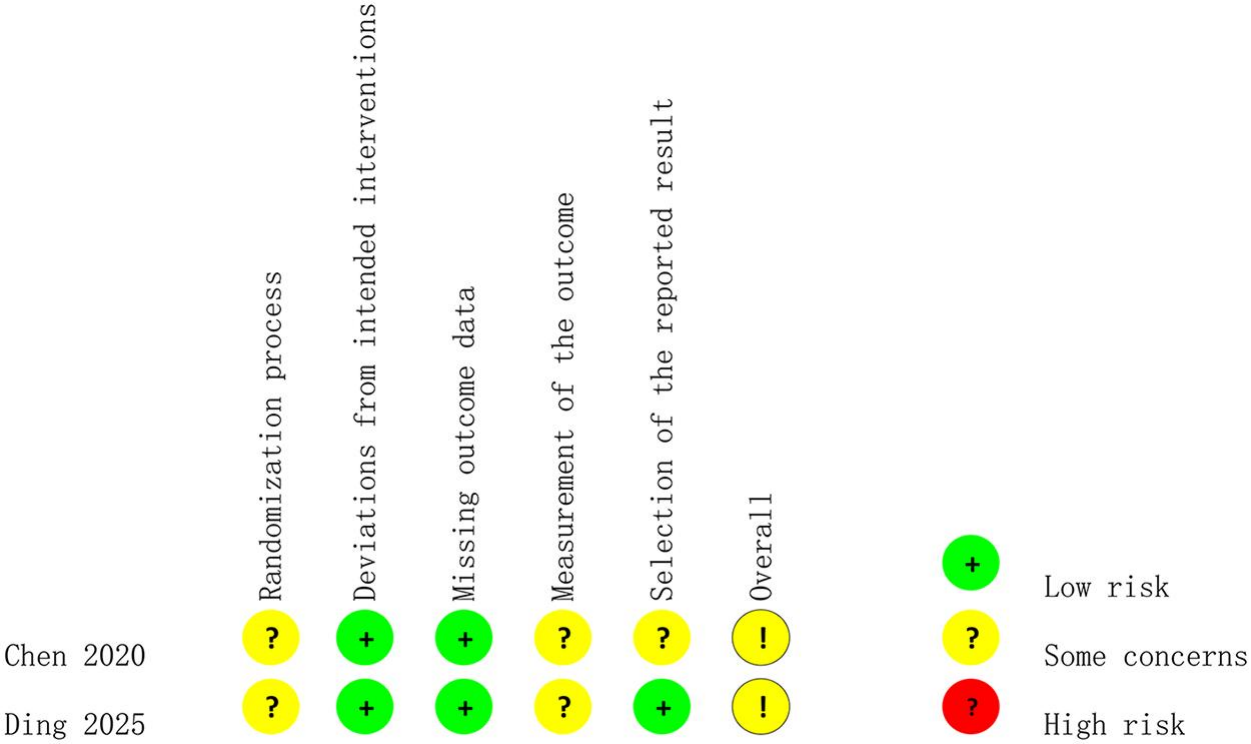
Risk of bias assessment of included RCTs using the RoB 2.0 tool.

**Figure 3:**
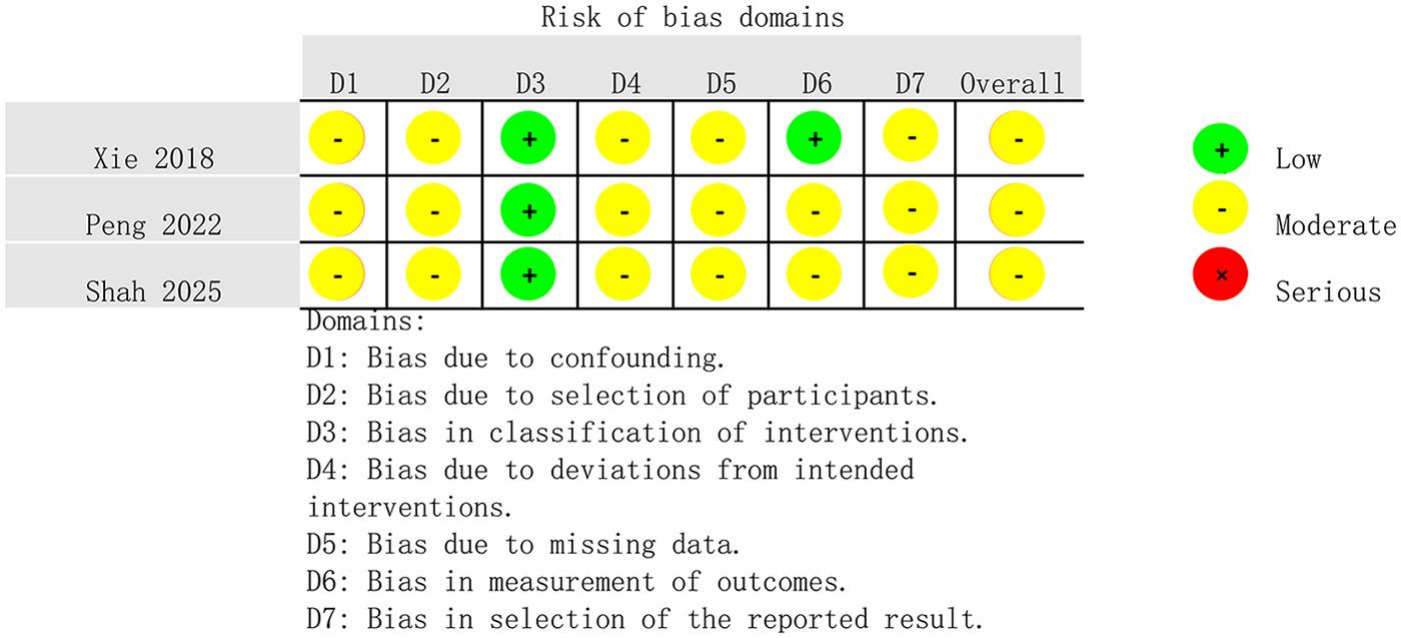
Risk of bias assessment of included cohort studies using the ROBINS-I tool.

A total of 2 RCTs and 3 cohort studies were included. The RoB 2.0 tool was used to assess the risk of bias for RCTs. This tool classifies the overall risk of bias into three levels, which are used to evaluate methodological flaws throughout the trial. Both included RCTs were rated as having some concerns, indicating that potential bias existed across the studies and that the reliability of their findings was limited. The risk of bias of the 3 cohort studies was assessed using the ROBINS-I tool, which grades bias into three categories. All cohort studies were rated as having a moderate risk of bias. This finding indicates that the studies had partial methodological limitations, and their conclusions should be interpreted with caution. The results of the methodological quality evaluation are shown in Figures 2, 3.

### 3.4 Meta-analysis of outcomes

#### 3.4.1 Surgical outcomes

**Figure 4:**
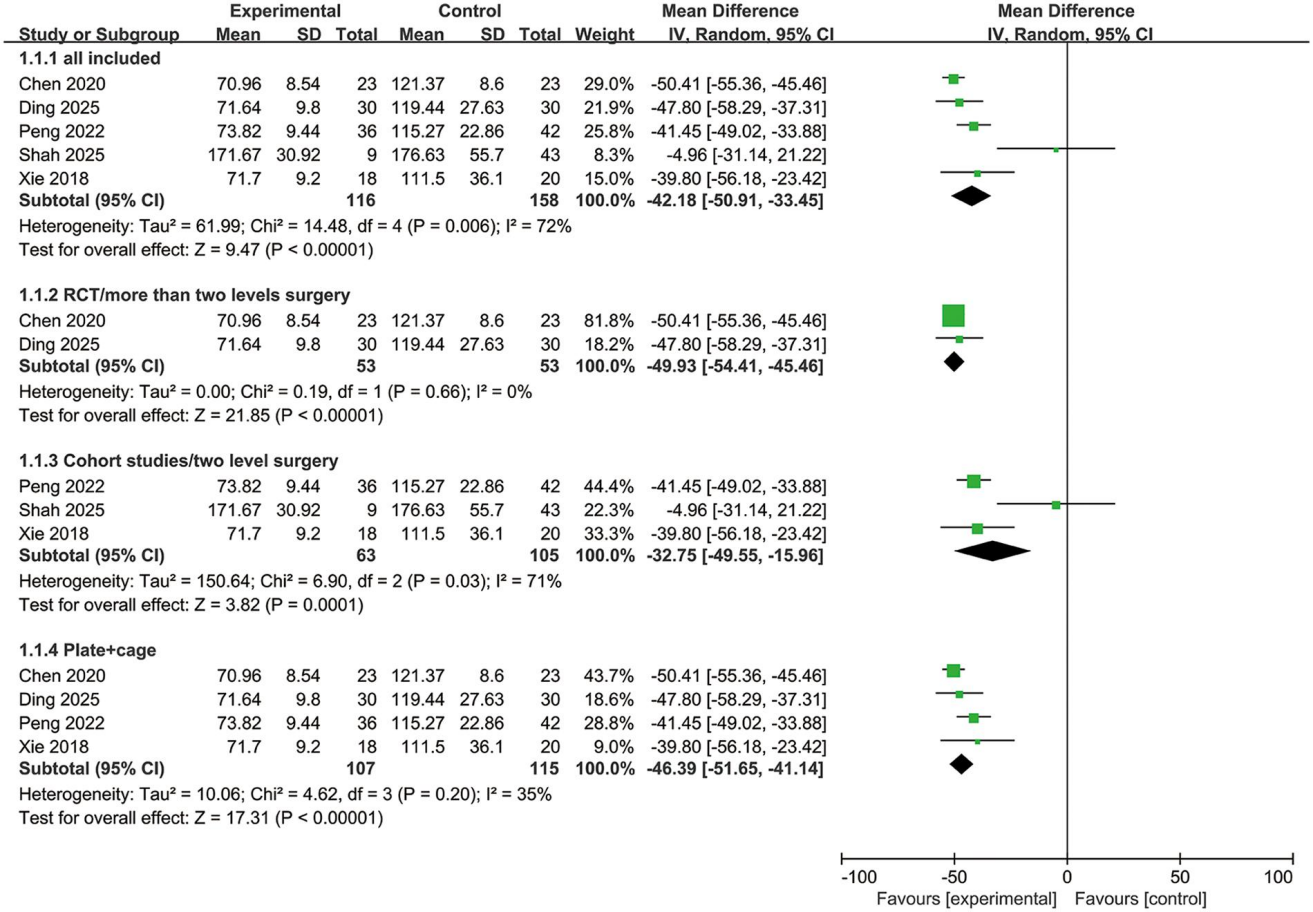
Comparison of surgical times.

**Figure 5:**
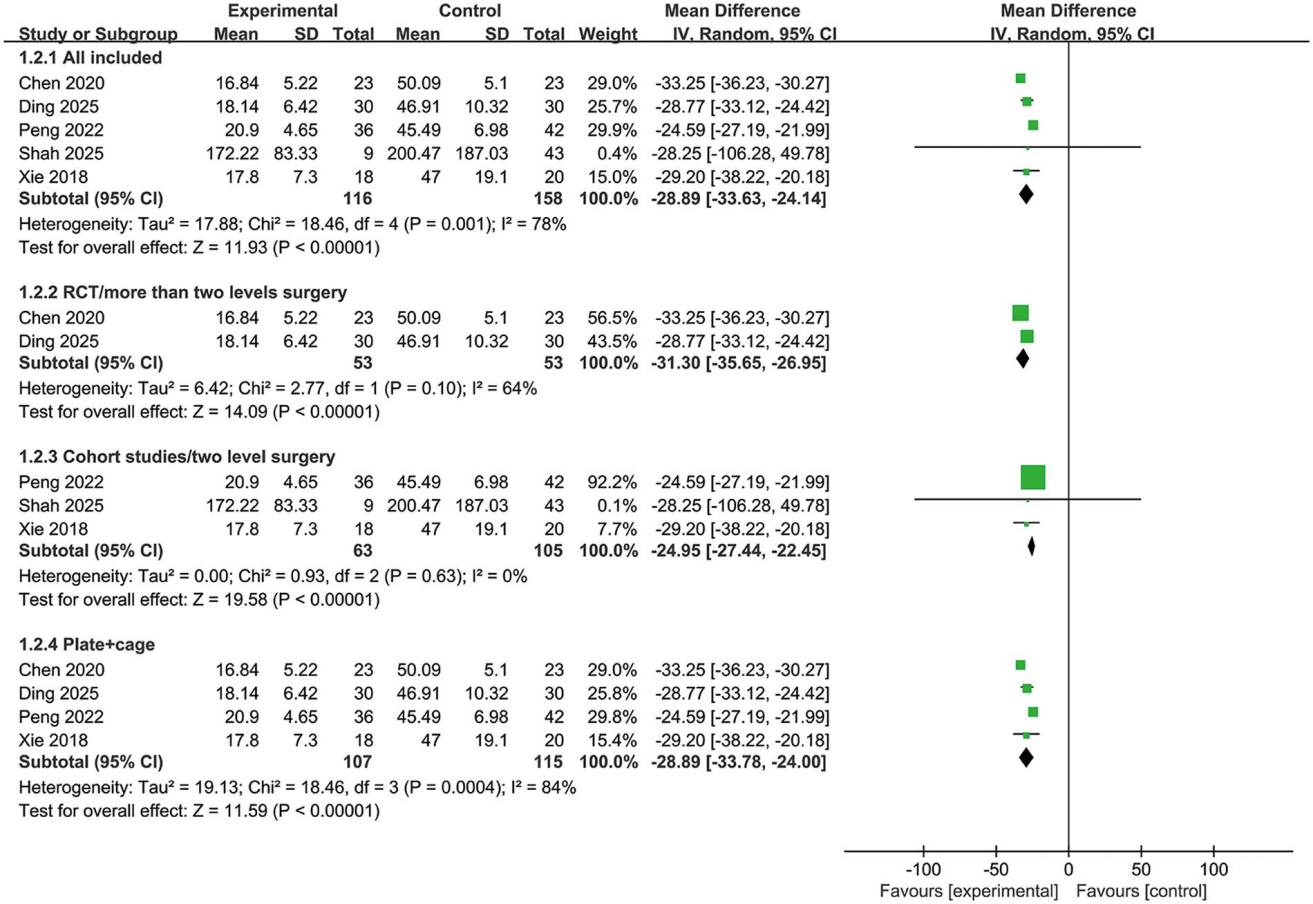
Comparison of intraoperative blood loss.

Five studies with 116 patients in the skip-level ACDF group and 158 patients in the contiguous ACDF group were included in the meta-analysis of surgical outcomes. The surgical time [MD: −42.18, 95% CI (−50.91, −33.45), I^2^ = 72%, Figure 4] and intraoperative blood loss [MD: −28.89, 95% CI (−33.63, −24.14), I^2^ = 78%, Figure 4] were significantly lower in the skip-level ACDF group than in the contiguous ACDF group. The surgical time in skip-level ACDF was significantly lower than that in contiguous ACDF in the subgroup analysis of RCTs or more than two-level surgery [MD: −49.93, 95% CI (−54.41, −45.46), I^2^ = 0%, Figure 4], subgroup analysis of cohort studies or two-level surgery [MD: −32.75, 95% CI (−49.55, −15.96), I^2^ = 71%, Figure 4] and subgroup analysis of plate and cage construction [MD: −46.39, 95% CI (−51.65, −41.14), I^2^ = 35%, Figure 4]. Intraoperative blood loss was significantly lower in skip-level ACDF than in contiguous ACDF in the subgroup analysis of RCTs or more than two-level surgery [MD: −31.30, 95% CI (−35.65, −26.95), I^2^ = 64%, Figure 5], subgroup analysis of cohort studies or two-level surgery [MD: −24.95, 95% CI (−27.44, −22.45), I^2^ = 0%, Figure 5] and subgroup analysis of plate and cage construction [MD: −28.89, 95% CI (−33.78, −24.00), I^2^ = 84%, Figure 5].

#### 3.4.2 Clinical outcomes

**Figure 6:**
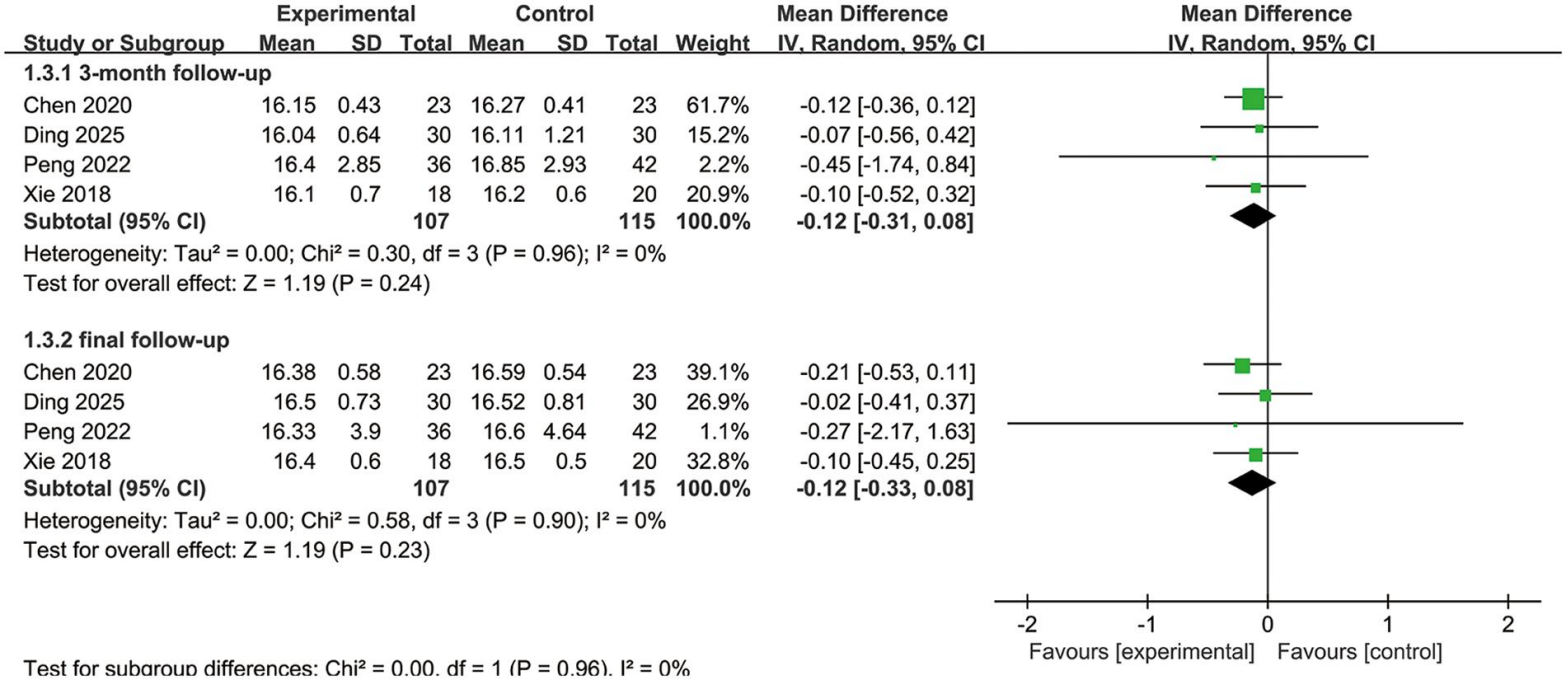
Comparison of JOA scores.

Four studies with 107 patients in the skip-level ACDF group and 115 patients in the contiguous ACDF group were included in the meta-analysis of the JOA score. No significant difference was found in the JOA score at the 3-month follow-up [MD: −0.12, 95% CI (−0.31, 0.08), I^2^ = 0%, Figure 6] or final follow-up [MD: −0.12, 95% CI (−0.33, 0.08), I^2^ = 0%, Figure 6] between the two groups.

#### 3.4.3 Radiological outcomes

**Figure 7:**
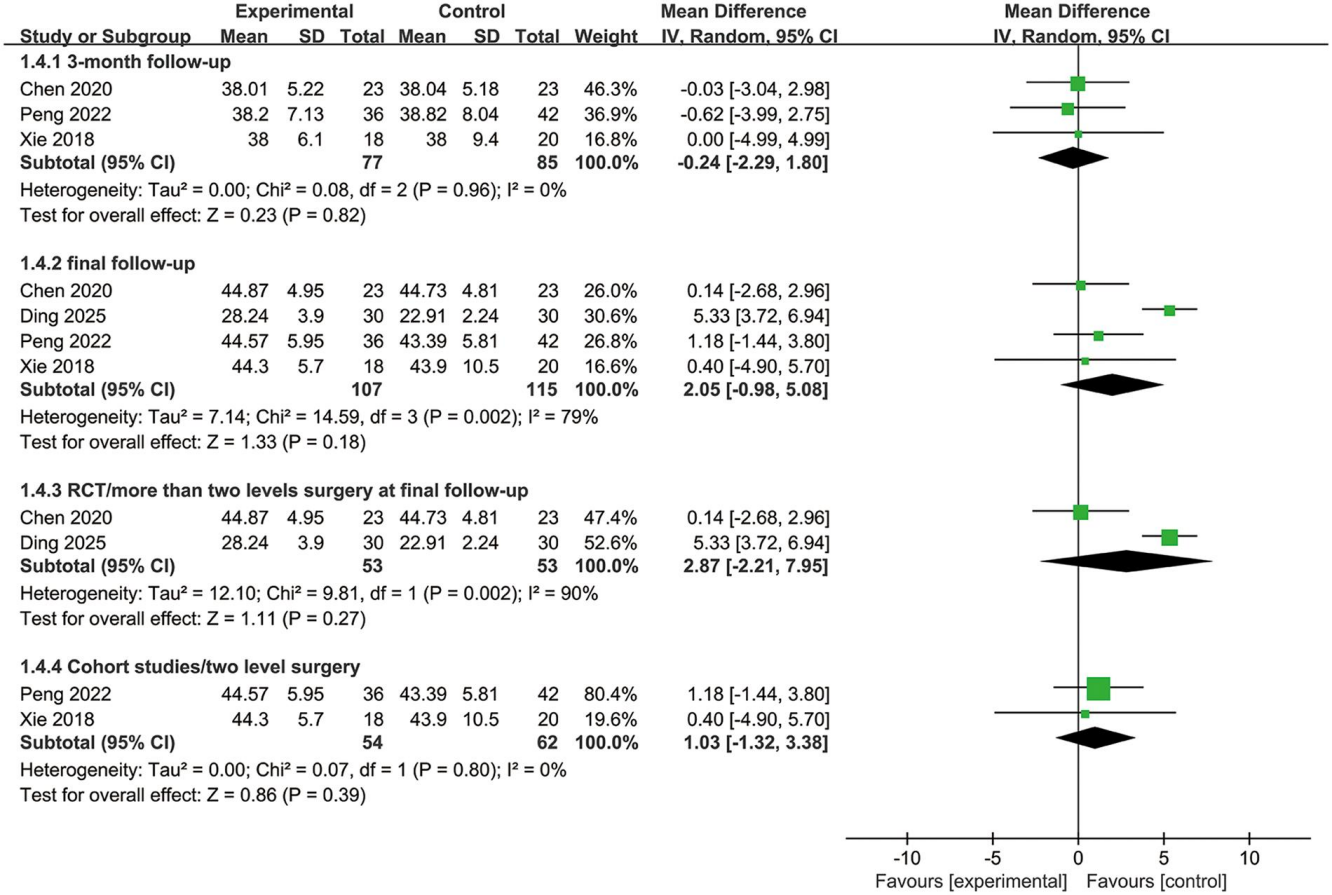
Comparison of C2-7 ROMs.

**Figure 8:**
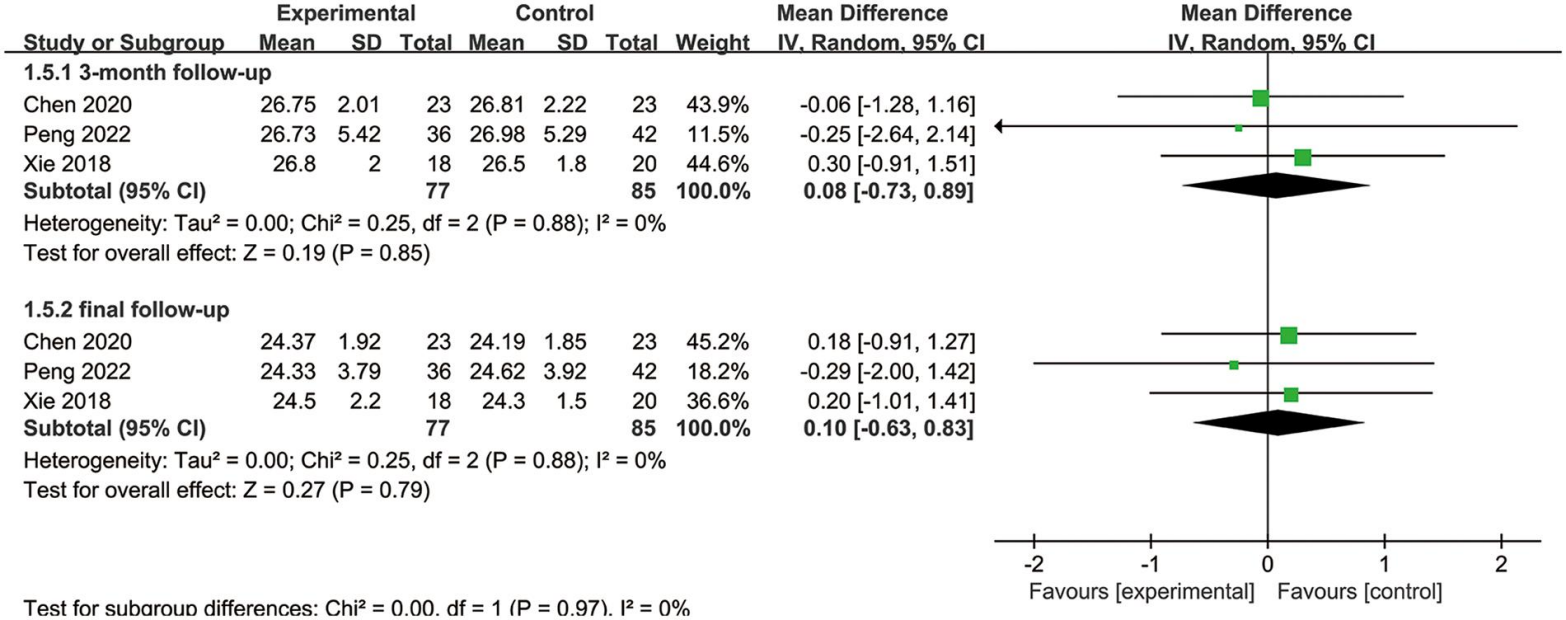
Comparison of the C2-7 SVA.

**Figure 9:**
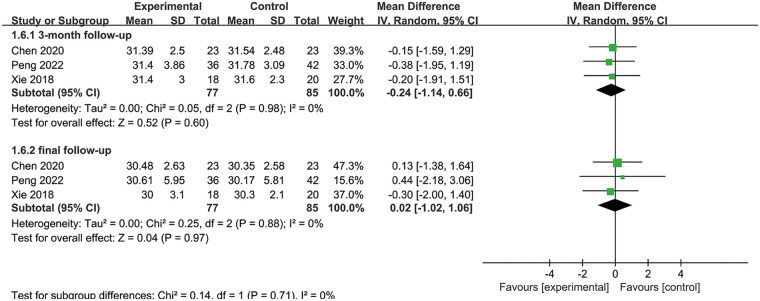
Comparison of the T1 slope.

Four studies with 107 patients in the skip-level ACDF group and 115 patients in the contiguous ACDF group were included in the meta-analysis of C2-7 ROM at the final follow-up. Three studies with 77 patients in the skip-level ACDF group and 85 patients in the contiguous ACDF group were included in the meta-analysis of C2-7 ROM at the 3-month follow-up, C2-7 SVA and the T1 slope. We found no significant difference in C2-7 ROM between the two groups at the 3-month follow-up [MD: −0.24, 95% CI (−2.29, 1.80), I^2^ = 0%, Figure 7] or at the final follow-up [MD: 2.05, 95% CI (−0.98, 5.08), I^2^ = 79%, Figure 7]. No significant difference in C2-7 ROM was detected between the subgroup analysis of RCTs or those with more than two-level surgeries [MD: 2.87, 95% CI (−2.21, 7.95), I^2^ = 90%, Figure 7], cohort studies or two-level surgeries [MD: 1.03, 95% CI (−1.32, 3.38), I^2^ = 0%, Figure 7] at the final follow-up. No significant difference in C2-7 SVA at the 3-month follow-up [MD: 0.08, 95% CI (−0.73, 0.89), I^2^ = 0%, Figure 8] or final follow-up [MD: 0.10, 95% CI (−0.63, 0.83), I^2^ = 0%, Figure 8] was detected between the two groups. No significant difference was detected between the two groups in terms of the T1 slope at the 3-month follow-up [MD: −0.24, 95% CI (−1.14, 0.66), I^2^ = 0%, Figure 9] or final follow-up [MD: 0.02, 95% CI (−1.02, 1.06), I^2^ = 0%, Figure 9].

**Figure 10:**
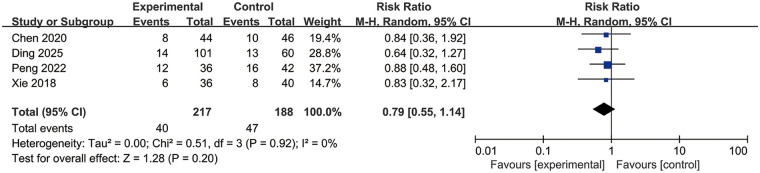
Comparison of adjacent segment degeneration.

Four studies with 217 segments in the skip-level ACDF group and 188 segments in the contiguous ACDF group were included in the meta-analysis of adjacent segment degeneration. No significant difference in adjacent segment degeneration [RR: 0.79; 95% CI (0.55, 1.14); I^2^ = 0%; Figure 10] was detected between the two groups.

#### 3.4.4 Complication rate

**Figure 11:**
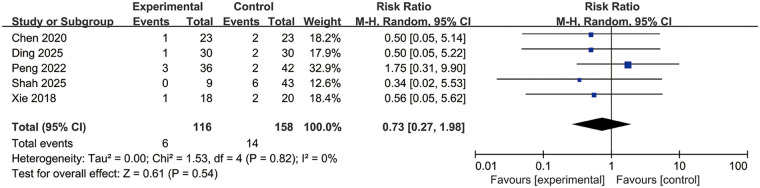
Comparison of complication rates.

Five studies with 116 patients in the skip-level ACDF group and 158 patients in the contiguous ACDF group were included in the meta-analysis of the complication rate. No significant difference in terms of complication rate was detected between the two groups [RR: 0.73, 95% CI (0.27, 1.98), I^2^ = 0%, Figure 11].

### 3.5 Meta-regression and publication bias assessment

Fewer than 10 studies were included; under these circumstances, meta-regression and funnel plots could not be constructed.

### 3.6 Sensitivity analysis and certainty of evidence

A sensitivity analysis was conducted to assess the stability of the results. No significant changes were observed in the results following the sequential exclusion of each study from the analysis. In this case, the results were reliable. The grade of the evidence of JOA score, C2-7 SVA, T1 slope, adjacent segment degeneration and complication rate were considered low and the grade of the evidence of surgical time, intraoperative blood loss and C2-7 ROM were considered very low (Supplementary Table 2).

## 4 Discussion

The optimal surgical strategy for skip-level CDD remains controversial. Continuous ACDF is associated with elevated rates of pseudarthrosis due to impaired osseous union. Conversely, skip-level ACDF preserves mobility at the intermediate segment and avoids the biomechanical consequences of long-segment arthrodesis; however, concerns persist regarding accelerated adjacent segment degeneration. We therefore performed a head-to-head comparison of these two approaches, assessing clinical outcomes, radiographic findings, and complication rates in patients who underwent surgical treatment for skip-level CDD. The results demonstrated that skip-level ACDF resulted in a shorter operative time and decreased intraoperative blood loss. No significant differences in the JOA score, C2-7 ROM, C2-7 SVA, T1 slope, complication rate or adjacent segment degeneration were detected between the two groups.

### 4.1 Surgical data

The results of our meta-analysis demonstrated that the operative time and intraoperative blood loss of skip-level ACDF were both significantly lower than those of contiguous ACDF. The surgical outcomes of one study (7) were significantly better than those of the other studies (24–27). Surgical techniques and clinical experience may account for the occurrence of this phenomenon. As reported by Baumann et al. (28), the surgical time of the learning phase was significantly greater than that of the competency phase concerning cervical spine surgery. Moreover, the number of intact intermediate segments of patients with skip-level CDD included in different studies varies, which leads to discrepancies in surgical exposure difficulty. This not only has a certain effect on the operative duration and amount of intraoperative blood loss but also may contribute to the occurrence of heterogeneity. Moreover, a shorter operative time and less intraoperative blood loss eventually lead to less surgical trauma and better recovery after surgery. However, the heterogeneity in operative time and intraoperative blood loss across the included studies was substantial, which may have compromised the reliability of the pooled results.

### 4.2 Complications

No significant difference in the complication rate was identified between the two groups. Five patients complained of dysphagia, and one patient complained of hoarseness in the skip-level ACDF group. Five patients complained of dysphagia, three patients complained of hoarseness, two patients complained of worsening symptoms, two patients experienced cage subsidence, and one patient needed reoperation because of a cervical hematoma in the contiguous ACDF group. The main complications of the two groups were dysphagia and hoarseness. Oh et al. (29) reported that the incidence of recurrent laryngeal nerve palsy was 1.2% in patients who underwent ACDF surgery. Gowd et al. (30) reported that hoarseness may not be equivalent to recurrent laryngeal nerve injury after ACDF. The number of surgeries was a risk factor for postoperative hoarseness caused by retraction to the laryngeal tissue. Moreover, the anterior cervical plates may irritate the anterior cervical soft tissue, resulting in dysphagia. Zhang et al. (13) compared zero-p anchored spacer and plate fixation for the treatment of skip-level CDD and reported that the dysphagia rate of the zero-p group was significantly lower than that of the anterior plate group, which indicated that zero-p implants may be a better choice for skip-level CDD.

### 4.3 Clinical outcomes

No significant difference in the JOA score was identified between the two groups. Baram et al. (10) retrospectively analyzed the results of 32 patients with skip-level CDD who underwent skip-level ACDF and reported that the mJOA score and NDI score significantly improved at the final follow-up. Sun et al. (31) conducted a randomized controlled trial comparing zero-p implants and cervical disc replacement for the treatment of skip-level CDD, with a mean follow-up of 32.4 months. They reported that the JOA score and NDI score significantly improved in both groups, but no significant difference was detected between the two groups. Both skip-level ACDF and contiguous ACDF are effective procedures for the treatment of CDD.

### 4.4 Radiological outcomes

Preservation of physiological cervical motion and sagittal alignment is widely recognized as a critical determinant of functional outcomes and patient satisfaction following cervical fusion surgery. Biomechanically, increasing the length of the fusion construct would logically be associated with greater restriction of global cervical mobility. Nevertheless, our pooled analysis revealed no significant difference in global C2-C7 ROM between patients who underwent skip-level ACDF and those who underwent contiguous ACDF for noncontiguous CDD. These findings can be attributed to the well-described compensatory mechanism whereby nonfused segments increase their motion to offset the loss of mobility at fused levels. In a validated C0–C7 finite element model, Liang et al. (32) reported that C5–C6 fusion resulted in a generalized increase in ROM across all nonfused segments in flexion, extension, lateral bending, and axial rotation, with corresponding elevations in intradiscal pressure and facet joint loading. Lebl et al. (33) confirmed these results in a human cadaveric study, further demonstrating that compensatory hypermobility is not limited to immediately adjacent segments but also occurs at more remote noncontiguous levels. A major limitation of the current evidence base, and consequently of our meta-analysis, is the exclusive reliance on global C2‒C7 ROM as the sole measure of cervical mobility. As correctly noted in the literature, global ROM provides only a superficial assessment of cervical spine function. The cervical spine operates through complex coupled motions, and its kinematic behavior is best evaluated at the segmental level. Most critically, none of the included studies assessed motion specifically at the preserved intermediate segment, the defining feature of skip-level ACDF. This means that we cannot determine whether the preserved intermediate segment exhibits normal kinematics or whether it develops compensatory hypermobility that could predispose patients to accelerated degeneration over time. Additionally, global ROM measurements cannot detect subtle changes in instantaneous axes of rotation or alterations in coupled motion patterns that may be functionally significant. Future biomechanical and clinical studies should employ advanced segmental motion analysis techniques to compare the kinematic profiles of skip-level and contiguous fusion, with a particular focus on the long-term behavior of the intermediate segment. Such investigations will be essential for fully understanding the biomechanical implications of intermediate segment preservation.

No significant difference in the T1 slope or C2-7 SVA was detected between the two groups. In recent years, spine surgeons have recognized that cervical sagittal imbalance is one of the causes of postoperative pain and dysfunction (34), and the issue of cervical sagittal balance in patients has attracted substantial attention. Li et al. (34) also demonstrated that adverse sagittal alignment was strongly associated with postoperative adjacent segment degeneration (*p* = 0.009). Li et al. (35) retrospectively reviewed 98 patients who underwent one-level ACDF and divided the patients into two groups on the basis of the occurrence of adjacent segment degeneration. They reported that adjacent segment degeneration was associated with a significantly lower T1 slope and sagittal segmental alignment and a greater preoperative spinal cranial angle. However, multivariate analysis revealed that the preoperative spinal cranial angle was the only independent risk factor for adjacent segment degeneration. Xie et al. (36) retrospectively analyzed 127 patients who underwent one-, two- and more than three-level ACDF. They reported that multilevel ACDF led to obvious loss of the C2–7 Cobb angle and that the T1 slope was significantly associated with postoperative loss of the C2–7 Cobb angle. Sagittal imbalance is a crucial predisposing factor for intervertebral disc degeneration in adjacent segments following multilevel cervical fusion surgery. An increase in the SVA distance elevated the stress burden of cervical fusion on adjacent segments, thereby accelerating the degeneration of these segments (37).

### 4.5 Adjacent segment degeneration

In the short-term follow-up period (predominantly 12–24 months) of the included studies, the pooled incidence of adjacent segment degeneration was 18.4% in the skip-level ACDF group and 25.0% in the contiguous ACDF group, with no statistically significant difference observed between the two groups. Notably, two included studies specifically reported the incidence of adjacent segment degeneration at the preserved intermediate segment in the skip-level cohort: Peng et al. (26) reported a 5.6% rate of intermediate segment degeneration, whereas Xie et al. (24) reported a slightly higher rate of 14.3%. These findings suggest that the preserved intermediate segment does not appear to undergo accelerated degeneration during the early postoperative period. Biomechanical studies have provided potential mechanistic explanations for these early clinical observations. Finn et al. (38) compared kinematic changes in human cervical spine specimens following skip-level and contiguous three-level ACDF. They reported that compared with intact spine, contiguous fusion resulted in a 72% increase in the ROM at the superior and inferior adjacent segments, whereas skip-level fusion led to only a 35% increase in the ROM at the adjacent and intermediate segments. This significant reduction in compensatory hypermobility may be attributed to preservation of the posterior ligamentous complex and increased physiological load distribution in skip-level constructs. Nouri et al. (39) extracted data from the Sanford Coordination of Rare Diseases at Sanford (CoRDS) and enrolled a total of 75 patients with Klippel-Feil syndrome, which arises from vertebral segmentation disorders during the embryonic development stage. In accordance with the Samartzis classification criteria, the subjects with cervical spine fusion were divided into Type Ⅰ, Type Ⅱ and Type Ⅲ groups. This study revealed that congenital noncontiguous fusion accounts for approximately 20% of Klippel-Feil deformities. Moreover, the ROMs of nonfused segments and the incidence of adjacent segment disease in these patients were significantly greater than those in the general population. Xu et al. (40) further demonstrated in a validated C2-7 finite element model that skip-level fusion with zero-profile anchored spacers resulted in significantly lower ROM, intradiscal pressure, and endplate stress at all nonfused segments than both skip-level and contiguous fusion using traditional anterior plates did. These preclinical data indicate that skip-level fusion, particularly with zero-profile devices, may confer a theoretical long-term advantage in reducing the risk of adjacent segment degeneration. Notably, all the clinical findings presented above are limited to short-term follow-up. As extensively documented in the literature, clinically significant adjacent segment degeneration is a late complication of cervical fusion surgery. The incidence of adjacent segment degeneration after cervical fusion varies considerably across studies, with reported overall rates ranging from 25% to 92% (41–44). Cumulative incidence rates increase progressively with follow-up, reaching 11%–12% at 5 years (45) and 16%–75% at 10 years post-operatively (4, 46). The 12–24 months follow-up duration of the included studies was insufficient to capture the full natural history of adjacent segment degeneration development. Therefore, our results cannot be extrapolated to conclude that skip-level fusion does not increase the long-term risk of adjacent segment degeneration. The theoretical biomechanical advantages observed in preclinical studies remain to be validated in large-scale, prospective clinical trials with at least 5–10 years of follow-up.

### 4.6 Limitations

This was the first systematic review and meta-analysis comparing skip-level ACDF vs. contiguous ACDF for skip-level CDD. However, several limitations should be mentioned: 1) Only five studies with 274 patients were included, as the limited evidence base substantially restricts the robustness of the pooled estimates and weakens the reliability of the conclusions; 2) the follow-up duration of the patients was relatively short: this duration is insufficient to properly evaluate long-term outcomes such as adjacent segment degeneration, which typically develops several years after ACDF; and 3) high heterogeneity was observed in key outcomes, particularly in terms of operative time, intraoperative blood loss and C2-7 ROM, and the number of skipped levels, number of surgeries, study design and implant type differed among the included studies, which might influence the results of this meta-analysis. Thus, more high-quality randomized controlled trials with long follow-up durations are needed to further compare skip-level ACDF and contiguous ACDF in the future.

## 5 Conclusion

Current evidence suggests that skip-level ACDF can reduce surgical time and intraoperative blood loss. Current evidence does not reveal a statistically significant increase in adjacent segment degeneration following skip-level ACDF. However, the available evidence remains limited.

## Data Availability

The original contributions presented in the study are included in the article/Supplementary Material, further inquiries can be directed to the corresponding author.
