# Supplementary material for: Comparison of skip-level ACDF vs. contiguous ACDF in the treatment of skip-level cervical degenerative disease: a systematic review and meta-analysis
Source: Front Surg. 2026 Jul 28;13:1817062. doi: 10.3389/fsurg.2026.1817062 (PMC13457221; doi:10.3389/fsurg.2026.1817062)
Supplement: Supplementary file 1 [file Table1.docx]

Supplementary Table 1 Search strategy

| Databases | Search No. | Index terms and Keywords |
| --- | --- | --- |
| PubMed | #1 | anterior cervical discectomy and fusion [Title/Abstract] OR ACDF [Title/Abstract] |
|  | #2 | Noncontiguous [Title/Abstract] OR skip level [Title/Abstract] OR skip-level [Title/Abstract] OR non-contiguous [Title/Abstract] OR noncontinuous [Title/Abstract] OR non-continuous [Title/Abstract] |
|  | #3 | #1 AND #2 |
| WOS | #1 | **TI=(**anterior cervical discectomy and fusion**) OR AB=(**anterior cervical discectomy and fusion**) OR TI=(**ACDF**) OR AB=(**ACDF**)** |
|  | #2 | **TI=(**Noncontiguous**) OR AB=(**Noncontiguous**) OR TI=(**Non-contiguous**) OR AB=(**Non-contiguous**)** |
|  | #3 | **TI=(**noncontinuous**) OR AB=(**noncontinuous**) OR TI=(**non-continuous**) OR AB=(**non-continuous**)** |
|  | #4 | **TI=(**skip level**) OR AB=(**skip level**) OR TI=(**skip-level**) OR AB=(** skip-level**)** |
|  | #5 | **#2 OR #3 OR #4** |
|  | #6 | #1 AND #5 |
| EMBASE (via Ovid) | 1 | anterior cervical discectomy and fusion.ab,ti. |
|  | 2 | ACDF.ab,ti. |
|  | 3 | Noncontiguous.ab,ti. |
|  | 4 | Non-contiguous.ab,ti. |
|  | 5 | skip level.ab,ti. |
|  | 6 | skip-level.ab,ti. |
|  | 7 | noncontinuous.ab,ti. |
|  | 8 | Non-continuous.ab,ti. |
|  | 9 | 1 or 2 |
|  | 10 | 3 or 4 or 5 or 6 or 7 or 8 |
|  | 11 | 9 and 10 |
| CNKI | 1 | SU=非连续 |
|  | 2 | SU=跳跃 |
|  | 3 | SU=融合 |
|  | 4 | SU=颈椎 |
|  | 5 | SU=前路 |
|  | 6 | 1 OR 2 |
|  | 7 | 3 AND 4 AND 5 AND 6 |
| Wanfang | 1 | 主题:(颈椎) |
|  | 2 | 主题:(融合) |
|  | 3 | 主题:(前路) |
|  | 4 | 主题:(跳跃) |
|  | 5 | 主题:(非连续) |
|  | 6 | 4 OR 5 |
|  | 7 | 1 AND 2 AND 3 AND 6 |
