# Supplementary material for: Comparison of skip-level ACDF vs. contiguous ACDF in the treatment of skip-level cervical degenerative disease: a systematic review and meta-analysis
Source: Front Surg. 2026 Jul 28;13:1817062. doi: 10.3389/fsurg.2026.1817062 (PMC13457221; doi:10.3389/fsurg.2026.1817062)
Supplement: Supplementary file 2 [file Table2.docx]

Supplementary Table 2 GRADE certainty grading evaluation.

| Outcomes | Certainty assessment | | | | | | | No. of participants | Certainty |
| --- | --- | --- | --- | --- | --- | --- | --- | --- | --- |
|  | **Number of Studies** | Study design | Risk of bias | Inconsistency | Indirectness | Imprecision | Other considerations |  |  |
| Surgical time | 5 | RCT/RCS | not serious | downgrade due to  I^2^ = 72% | not serious | not serious | none | 274 | ⨁◯◯◯  Very low |
| Intraoperative blood loss | 5 | RCT/RCS | not serious | downgrade due to  I^2^ = 78% | not serious | not serious | none | 274 | ⨁◯◯◯  Very low |
| JOA score | 4 | RCT/RCS | not serious | not serious | not serious | not serious | none | 222 | ⨁⨁◯◯  Low |
| C2-7 ROM | 4 | RCT/RCS | not serious | downgrade due to  I^2^ = 79% | not serious | not serious | none | 222 | ⨁◯◯◯  Very low |
| C2-7 SVA | 3 | RCT/RCS | not serious | not serious | not serious | not serious | none | 162 | ⨁⨁◯◯  Low |
| T1 slope | 3 | RCT/RCS | not serious | not serious | not serious | not serious | none | 162 | ⨁⨁◯◯  Low |
| Adjacent segment degeneration | 4 | RCT/RCS | not serious | not serious | not serious | not serious | none | 405 | ⨁⨁◯◯  Low |
| Complication rate | 5 | RCT/RCS | not serious | not serious | not serious | not serious | none | 274 | ⨁⨁◯◯  low |

Note: RCS retrospective cohort study; RCT randomized controlled trial.
